# Supplementary material for: A Whole-Genome Sequencing-Based Approach for the Characterization of Klebsiella pneumoniae Co-Producing KPC and OXA-48-like Carbapenemases Circulating in Sardinia, Italy
Source: Microorganisms. 2023 Sep 20;11(9):2354. doi: 10.3390/microorganisms11092354 (PMC10535212; doi:10.3390/microorganisms11092354)
Supplement: Supplementary file 1 [file microorganisms-11-02354-s001.zip › supplementary Table_S1.pdf]

Table S1: Plasmids identified and resistance genes carried

Homologous plasmid (Genbank acc. n°)	N° of strains (%)	Total resistance genes identified
Klebsiella pneumoniae plasmid pGMI16-005_01 (CP028181)	17/17 (100)	aatA1, KPC3/31, OXA-9, TEM-122
Klebsiella pneumoniae plasmid pCMY2_085072 (CP028804)	17/17 (100)	AAC(6')-Ib9, aadA2.APH(3')-Ia, APH(3'')-Ib, APH(3')-VI,APH(6)-Id, CMY-59, dfrA12, floR, mphA, OXA-9, sul1, sul2, TEM-122, tet(A)
Shigella sp. plasmid pLN126_33(HE578058)	17/17 (100)	-
Klebsiella pneumoniae plasmid pKpQIL-SC29 (NC_021656)	17/17 (100)	-
Salmonella enterica plasmid pSQR1 (KJ187750)	11/17 (64.7)	OXA-181, qnrS1
Klebsiella pneumoniae plasmid pKpN06-NDM7 (CP012995)	9/17 (52.9)	AAC(6')-Ib9, ANT(3'')-Iia, APH(3')-Ia, arr-2, cmlA5, dfrA14, OXA-9, OXA-10, OXA-181, qnrS1, TEM-122
Klebsiella pneumoniae plasmid pUCLAOXA232-2 (CP012563)	5/17 (29.4)	AAC(6')-Ib9, ANT(3'')-Iia, APH(3')-Ia, arr-2, cmlA5, dfrA14, OXA-9, OXA-10, TEM-122
Salmonella enterica plasmid pHLR25 (NC_019110)	5/17 (29.4)	OXA-181, qnrS1
Klebsiella pneumoniae plasmid pIncX3 (CP035910)	2/17 (11.8)	ANT(3'')-Iia, APH(3')-Ia, arr-2, cmlA5, dfrA14, OXA-9, OXA-10, TEM-122
Klebsiella variicola strain plasmid p2_020019 (CP028550)	2/17 (11.8)	aadA2, APH(3'')-Ib, APH(3')-VI, APH(6)-Id, CMY-59, dfrA12, floR, mphA, sul1, sul2, TEM-122, tet(A)
Klebsiella pneumoniae strain AR_0075 plasmid unnamed5 (CP032190)	1/17 (5.9)	AAC(6')-Ib9, APH(3')-Ia, arr-2, cmlA5, dfrA14, OXA-10, TEM-122
