# Supplementary figures and images for: A Whole-Genome Sequencing-Based Approach for the Characterization of Klebsiella pneumoniae Co-Producing KPC and OXA-48-like Carbapenemases Circulating in Sardinia, Italy
Source: Microorganisms. 2023 Sep 20;11(9):2354. doi: 10.3390/microorganisms11092354 (PMC10535212; doi:10.3390/microorganisms11092354)

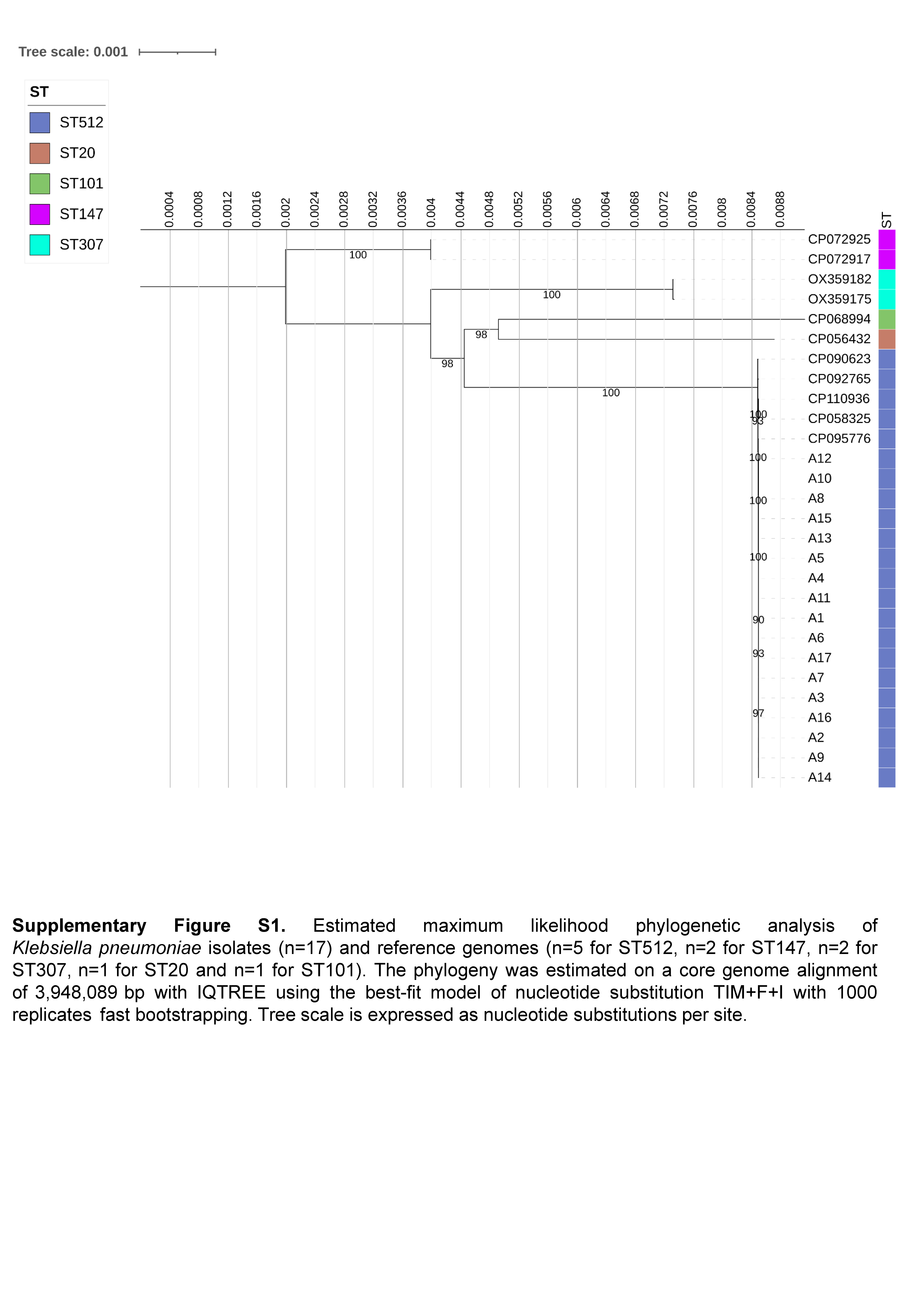

Supplement: Supplementary file 1 [file microorganisms-11-02354-s001.zip › Supplementary_figure_S1.tif]
